# Dynamic Robot Navigation in Confined Indoor Environment: Unleashing the Perceptron-Q Learning Fusion

**DOI:** 10.3390/s25206384

**Published:** 2025-10-16

**Authors:** M. Denesh Babu, C. Maheswari, B. Meenakshi Priya

**Affiliations:** 1 Department of Electronics and Communication Engineering, The Kavery Engineering College, Salem 636453, India; 2 Department of Mechatronics Engineering, Kongu Engineering College, Erode 638060, India; maheswari@kongu.ac.in (C.M.); bmp@kongu.ac.in (B.M.P.)

**Keywords:** global path planning, goal-seeking motion, reward function, robot navigation steering control, Threshold distance, vector angle

## Abstract

Robot navigation in confined spaces has gained popularity in recent years, but offline planning assumes static obstacles, which limits its application to online path-planning. Several methods have been introduced to perform an efficient robot navigation process. However, various existing methods mainly depend on pre-defined maps and struggle in a dynamic environment. Also, diminishing the moving costs and detour percentages is important for real-world scenarios of robot navigation systems. Thus, this study proposes a novel perceptron-Q learning fusion (PQLF) model for Robot Navigation to address the aforementioned difficulties. The proposed model is a combination of perceptron learning and Q-learning for enhancing the robot navigation process. The robot uses the sensors to dynamically determine the distances of nearby, intermediate, and distant obstacles during local path-planning. These details are sent to the robot’s PQLF Model-based navigation controller, which acts as an agent in a Markov Decision Process (MDP) and makes effective decisions making. Thus, it is possible to express the Dynamic Robot Navigation in a Confined Indoor Environment as an MDP. The simulation results show that the proposed work outperforms other existing methods by attaining a reduced moving cost of 1.1 and a detour percentage of 7.8%. This demonstrates the superiority of the proposed model in robot navigation systems.

## 1. Introduction

In robotics, one of the basic issues is PATH planning. Using a robot and an environment description, it might be stated as follows: create a path that avoids conflicts between the designated start and goal locations. The two classic forms are online planning, which focuses on dealing with active barriers and moderately recognized surrounds, and offline planning, which accepts static impediments and fully known settings [1]. Traditional offline planning methods [2] are inapplicable to online path-planning challenges because they presume static barriers. One strategy is to construct an initial path and utilize re-planning whenever it is not feasible to carry it out [3]. However, because dynamic barriers’ motion information is lacking, re-planning will be inefficient in terms of time (frequent re-planning) and path (oscillating motions and diversions). Moreover, robot deadlocks can cause re-planning tactics to fail. Some strategies use an additional temporal dimension in place of re-planning in order to prevent such conflicts [4]. This method, however, necessitates the knowledge of the future routes of active impediments and expands the number of states to be explored. One may try to describe the behavior of dynamic barriers and forecast their courses if the future motions of obstacles are uncertain [5]. However, the “freezing robot” problem may arise if the navigation task is divided into separate prediction and planning processes. The robot will thus be unable to discover any practical course of action since the anticipated routes may designate a significant area of the area as impassable.

Learning-based control paradigms for energy management issues have been developed in recent years thanks to artificial intelligence. By adaptively learning the interaction experience, reinforcement learning (RL), a model-free machine learning algorithm, builds the agent–environment interactions as a Markov decision process (MDP), maximizes the cumulative reward expectation, and ultimately resolves challenging decision-making issues. This was made popular by the ground-breaking study [6], which used deep neural networks to estimate the function of value-based RL. RL’s influence on the path-planning difficulty is currently hindered by a number of issues, despite the fact that it has shown exceptional performance in several areas. The reward becomes scarce in an enormously vast environment, which increases training effort and renders the learning process inefficient overall. The over-fitting problem presents another difficulty [7,8]. The robot’s generalizability to unfamiliar surroundings is low, and it is frequently restricted to training conditions. This has led to a great deal of interest in RL-based energy management strategies. In order to address the issue of nonspontaneous and flexible startup in demand response problems for residential and small commercial buildings, ref. [9] uses Q-learning (QL) to construct a completely automated energy management system. For real-time energy scheduling of electric car charging stations, a Hyperopia Sarsa-based RL algorithm is employed, which yields higher profits than the truncated sample-average approximation method [10]. In a similar vein, a QL-based approach to energy management was used in practice for a number of smart facilities in a price demand response setting. The conventional RL algorithms, namely QL and Sarsa, store state-action values in tables even if their convergence is almost assured. Large amounts of storage space are needed, and high-dimensional state-action issues are difficult for the algorithms to handle. Due to the exponential growth of the robot state, joint action, and joint opinion spaces with the number of robots [11], the bulk of current approaches continue to face scaling issues to arbitrarily large multi-robot systems [12]. As a result, the efficacy, generalizability, and scalability of existing RL-based organizers remain insufficient to suit the needs of a variety of presentations.

To address high-dimensional state decision-making challenges, researchers have combined deep reinforcement learning (DRL) with RL-based deep learning (DL). DL has strong information-awareness skills, and its strong function-fitting ability may be used to tackle the state-dimensional catastrophe problem [13]. Deep Q-network (DQN) is a conventional continuous state technique that uses a strong end-to-end learning capability to address high-dimensional state management issues. A multi-agent Bayesian DQN approach was utilized to regulate microgrid energy when communication failures resulted in information loss. A multi-buffer, double DQN based on convolutional neural networks is introduced, demonstrating impressive resilience enhancement and prompt reaction to changing energy system conditions. The mentioned works, however, concentrate on distinct behaviors. In recent years, a more potent deep deterministic policy gradient (DDPG) [14] has been frequently employed to address the issues raised by continuous action energy management. In order to reduce the energy expenses of smart houses, ventilation and energy storage systems (ESSs) were scheduled using an effective energy management algorithm based on DDPG [15]. Computer vision and DDPG, which can automatically determine the best control strategy from visual inputs, were used to increase the fuel efficiency of hybrid electric automobiles. To control home energy use in situations when load needs are unknown and information on electricity prices is scarce, a novel actor–critic DRL algorithm based on reward shaping was created.

Modern systems cannot have simply discrete or continuous control, even though the aforementioned DRL approaches are advised for energy management [16]. Furthermore, even though the DRL approach holds great promise for improving energy management, it has a flaw in that when the agent uses empirical data to optimize its policy, it will mistakenly believe that the high-value action is the best course of action because the state-action value is overestimated. This will cause the learning direction to veer off course and ultimately fall into a local optimum.

### Motivation

Dynamic robot navigation in constrained indoor spaces is a crucial issue with important real-world applications, including rescue operations, healthcare, and warehouse automation. These settings frequently provide unforeseen changes, such as shifting obstructions, restricted corridors, and poor sight, rendering typical path-planning and navigation approaches ineffective. Robots must function effectively in such environments while responding to dynamic aspects in real time, assuring safety and dependability. High-dimensional state and action spaces, sensor reading errors, and computer resource limits all add to the difficulty. Overcoming these obstacles requires novel ways that combine enhanced vision, decision-making, and control strategies to allow robots to navigate complex interior settings independently and efficiently. Recently, reinforcement learning techniques have become more popular in autonomous navigation systems. This technique has the ability to learn optimal navigation policies by understanding the environment and obtaining rewards without the presence of a pre-defined map. Thus, it motivates us to utilize the reinforcement learning concept in this work for an efficient navigation process. The main contributions of the research are as follows:

- To propose a new perceptron-Q learning fusion (PQLF) model for effective robot navigation in dynamic environments. The new combination of perceptron-based intelligence and the Q-reinforcement learning model allows the robots to make accurate decisions by avoiding obstacles.
- To offer an efficient reward system, a novel reward system is introduced, where a larger negative reward is assigned while the robots face an obstacle. Also, a small negative reward is assigned when it deviates from the goal, and a large positive reward is allocated for reaching the goal.
- To validate the strength of the proposed work, comprehensive experiments are performed, and the results are compared with other existing methods.

The rest of the paper is sorted as follows: Section 2 signifies the recent related works in the field of robotics navigation. Section 3 provides the proposed methodology, which includes the explanation of the proposed work and necessary equations; Section 4 provides the results analysis with a detailed comparison of proposed and existing models; Section 5 deals with the conclusion and future scope of the proposed work.

## 2. Related Works

The Brazilian mining company Vale S.A., (Rio de Janeiro, Brazil), developed the EspeleoRobo, a robotic platform, to inspect confined areas. Several localization and navigation control methods built into this platform are examined by Rezende et al. [17]. The posture estimation techniques based on wheel, optical, and LiDAR odometry and Ultra-Wideband radio signals were compared after being fused with IMU (Inertial Measurement Unit) data. Both autonomous and tele-operated robot operations are examined in the tests. An artificial vector field controller served as the foundation for the robot’s autonomous navigation, using the various posture estimations as input to steer the robot along predetermined routes. However, robot mobility is limited in confined environments due to their constrained spaces and small routes.

In order to learn policies that can dynamically adjust to the occurrence of moving pedestrians while traveling among desirable sites in limited situations, Pérez-D’Arpino et al. [18] suggested a reinforcement learning (RL)-based method. A motion planner provides waypoints for the policy network to follow a globally planned trajectory, while RL controls the local interactions. The investigation of a compositional basis for multi-layout training was demonstrated by the successful generalization of policies learned in a small number of geometrically simple layouts to more complex unseen configurations that display the composition of the structural components accessible during training. However, learning-based models may become less successful in practical applications if they overfit certain training datasets.

In the Robot Operating System (ROS), Ren et al. [19] create a chassis simulation environment and develop a novel navigation algorithm. First, an experimental framework was developed for a chassis navigation system based on new hardware and sensors. Second, the Extended Kalman Filter (EKF) is employed to fuse the odometer and inertial steering data. How the environmental map is made depends on the map’s construction methodology. The global path is planned using the A* method, and the local path is scheduled using the Dynamic Window Method (DWA). However, robots struggle to predict and adapt to the movement of dynamic obstacles, unlike humans and other robots.

The technique identified and avoided a variety of environmental barriers using an autonomous robot outfitted with stereo-vision cameras and ultrasonic rangefinder sensors. Based on an ANN, Singh et al. [20] introduced the Adaptive Squashing Function, which enables the best possible steering in a social environment with dynamic obstacles for active SLAM. The robot created a map of the surroundings and navigated the whole terrain using a trained artificial neural network based on an adaptive squashing function. For fifteen distinct cases with varying starting positions, the suggested method was contrasted with an exhaustive search and a random approach. However, it will be difficult to make decisions in real time when there are moving items around, such as people, other robots, or trolleys.

De Oliveira Júnior et al. [21] provided a method to improve the estimation of a wheeled mobile robot’s indoor position in an environment. The localization system integrates the Adaptive Monte Carlo Localization (AMCL) algorithm with location information and alterations based on artificial vision recognition of fiducial markers scattered across the environment to reduce the errors produced by the AMCL position estimation. The Robot Operating System (ROS) served as the foundation for the methodology, which was tried and proven in a simulation setting. However, a substantial amount of computing power is needed for obstacle recognition, course re-planning, and real-time sensor data processing.

Ren et al. [22] presented a reliable 3D robotic mapping and navigation technique designed especially for these settings. This technique creates accurate 3D maps by combining neural networks, simultaneous localization and mapping, and light detection and ranging. Additionally, it enhanced navigation and obstacle avoidance in dynamic and complex construction environments by combining deep reinforcement learning with grid-based pathfinding. The effectiveness of the approach is confirmed by an evaluation carried out in a simulated attic environment, which is marked by a variety of truss systems and constantly shifting obstructions. In addition to achieving over 95% mapping accuracy, this technology enhances navigation accuracy by 10% and increases efficiency and safety margins by more than 30% when compared to existing benchmarks.

Cai et al. [23] analyzed the enhanced particle swarm optimization (IPSO) and enhanced whale optimization algorithm (IWOA) techniques, evaluating their adaptability to various pollutants and their capacity to detect both constant and variable PM sources. The findings indicate that both approaches locate consistent PM sources with a 73.3% success rate, with IPSO providing a little higher efficiency. Furthermore, the approaches demonstrated consistent performance across a range of contaminants, with greater success rates in jobs requiring ethanol vapor. The complexity of PM dispersion is the main cause of this consistency, as well as the observed differences in success rates.

De Heuvel et al. [24] provided a spatiotemporal attention pipeline that uses 2D lidar sensor data to improve navigation. In addition to the suggested pipeline, new dynamic barriers are prioritized over static ones in this lidar-state representation. The attention process thus makes it possible to perceive scenes selectively in both space and time, which enhances navigation performance in dynamic situations. The method is tested in various simulators and settings and discovered that it had good generalization to unknown surroundings.

### Problem Statement

Limited interior conditions present autonomous robots with significant navigational issues due to their dynamic nature. These settings frequently have small paths, unexpected moving impediments, and complicated layouts, forcing robots to make real-time judgments while remaining safe and efficient. Existing navigation algorithms frequently struggle with high-dimensional state and action spaces, low sensor reliability, and the necessity for precise control in restricted environments. To solve these issues, a robust and adaptive navigation system that can handle uncertainties, optimize path planning, and dynamically respond to environmental changes in constrained interior areas is critical.

## 3. Proposed Methodology

Robot navigation in confined indoor environments has gained a lot of attention over the last few years. In the autonomous navigation system, a mobile robot must be able to assess its surroundings, avoid obstacles, plan a route from one place to another, and effectively regulate its direction in order to reach its destination. There are two conventional approaches to robot navigation: (i) offline planning, which accepts static difficulties and completely known surroundings, and (ii) planning, which focuses on dynamic impediments and partially known environments. Confined indoor spaces, such as offices, hospitals, or warehouses, involve narrow pathways, sharp turns, and dynamic obstacles (e.g., humans and other robots). For robots to remain efficient and prevent collisions, they must make judgments in real time. Hence, typical offline planning algorithms cannot be used directly to solve online path-planning tasks in constrained indoor environments because they assume that the barriers are static. Learning-based strategies have recently been investigated for online planning in dynamic environments. Specifically, RL has established unresolved performance in many presentations.

**Figure 1:**
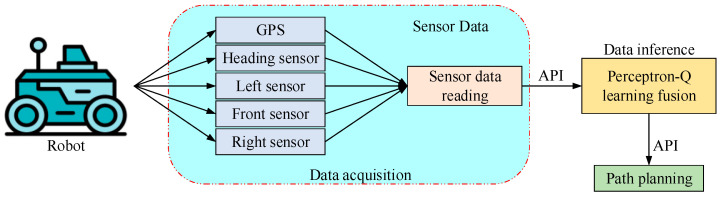
Block diagram of the proposed robot navigation system.

To overcome the above tasks, a new perceptron-Q learning fusion (PQLF) model is introduced in this study for Robot Navigation. For this, a generic three-wheeled robot (TWR) model with two front and passive omni wheels in the hind is employed. Three sensors are positioned around the robot’s left, right, and front sides, respectively, and a GPS module is positioned in the middle of the device. The global navigation controller receives data from GPS. Likewise, the perceptron-Q learning fusion model is fed by ultrasonic sensors. Before deciding which way to move, the robot must first determine whether or not there are any obstacles below the threshold. During local path-planning, the sensors provide the robot with information about the distances of close, mid, and far obstacles. The PQLF Model-based navigation controller of the robot will receive these details as input and function as an agent. The agent wanted to exploit the cumulative probable reward, which is the total of all payouts over time. The Dynamic Robot Navigation in Confined Indoor Surroundings can thus be expressed as an MDP. Figure 1 depicts the workflow diagram of the proposed framework.

For this study, a generic TWR model with two front and passive omni wheels in the hind is employed. Three sensors are positioned around the robot’s left, right, and front sides, respectively, and a GPS module is positioned in the middle of the device. The global navigation controller receives GPS data at each time interval (*γ*_*GPS*_). Likewise, the PQLF module receives input from ultrasonic sensors at each time intermission *γ*_*S*_, where *γ*_*S*_ is always equal to *γ*_*GPS*_.

### 3.1. Navigation Control

The robot must first determine whether or not there are any impediments below the threshold before determining which way to move. During local path-planning, the sensors provide the robot with information about the distances of close, mid, and far obstacles. It should be noted that the data from the sensors is ambiguous and inaccurate. Furthermore, language variables cannot be represented by traditional logical systems. Although these variables are frequently utilized in daily lives and are relatively simple to use, mathematical modeling of them may be difficult as well. Although humans often do not need exact mathematical or logical calculations, they are very adept at controlling extremely complicated systems. In this work, a PQLF model for Robot Navigation is presented.

PQLF handles the wheeled mobile robot’s local path-planning. Three ultrasonic sensors are mounted on the robot’s flanks to calculate the distance between objects on each side, allowing for easier local navigation. To avoid collisions, sensors are placed on the robot’s left, middle, and right parts, covering the semi-circular front. Sensor data is divided into three categories: frontal, left, and right. The goal is to arrive at the terminus without encountering any obstacles in strange surroundings.

Robot motion is separated into two categories: goal-seeking motion planning and obstacle avoidance. A goal-seeking algorithm is used to determine the direction to the destination during goal-seeking motion planning; as a result, steering and global route planning are modified appropriately. Otherwise, PQLF assumes steering control anytime an impediment is within the robot’s near range. In this case, 20 cm is set as the threshold value for any distance sensor. Robot position/orientation, target coordinates, and sensor data define the states of the environment’s MDP model.

### 3.2. Proposed Robot Navigation Using Perceptron-Q Learning Fusion Model

The proposed study utilized a new PQLF model for robot navigation by dynamically adjusting the rewards and thereby improving the adaptability of robots in an unseen environment. The proposed PQLF is a combination of MLP and Q-reinforcement learning. Integrating perceptron-based intelligence and the Q-reinforcement learning model helps the robots to make accurate predictions and effectively avoid obstacles.

RL is a kind of ML that incorporates agent interaction and improves the accumulation of rewards for actions performed in the environment. The MDP uses dynamic programming techniques in machine learning to determine the best course of action for maximizing rewards over time. In the proposed work, the reinforcement agent is considered an autonomous robot, which involves sensors for sensing the surroundings. The agents understand the surrounding information through Q-learning as per the robot’s future state, and the reward function is attained from present actions like stopping, turning right, turning left, stopping, and moving forward. The fundamental activities that RL performs are as follows:

- First, the agent interacts directly with the environment in each of its states to gather input.
- Second, the environment responds by giving positive or negative rewards for the activity, denoted by K+ or K−, respectively.
- Third, the agent maximizes the rewards that have already been gathered and recognizes changes in the surroundings.
- Fourth, starting from the current condition, the RL approach will be used at this point to increase the predicted reward rate.

In the proposed model, an efficient reward system is introduced, where a larger negative reward is allocated when the robots encounter an obstacle. Also, a small negative reward is assigned when it deviates from the goal, and a large positive reward is allocated while reaching the goal. The value iteration notion is used by Q learning, a reinforcement learning approach, where the agent evaluates the value function to update all states and actions for all repetitions in order to determine which action C results in the largest reward K. It can deal with the stochastic rewards in a non-adaptive way and is model-free. Essentially, in a state E, the agent performs an action C, records its reward K and the subsequent state $E^{\prime }$, and calculates the Q-value using Equation (1), where $C_{j}$, $E_{j}$ and $K_{j}$ represents the action, state, and reward at the time j, correspondingly 0<β<1 represents the learning rate and 0<δ<1 represents the absolute value of rewards.

(1) $$Q^{+}\left(E_{j},\;C_{j}\right)\leftarrow \left(1-\beta \right)Q^{+}\left(E_{j},\;C_{j}\right)+\beta \left(K_{j}+\delta_{\max}Q^{+}\left(E_{j},\;C_{j}\right)\right)$$

The proposed model utilizes two action selections based on the present policy. Also, the present policy will estimate future actions and new states and is stored in the Q-table. It is crucial to note that one of the reasons Q-learning is chosen as one of the RL techniques is that it is model-free. In addition, Q-learning may be used to approach stochastic rewards in a non-adaptive way. Moreover, Q-learning is capable of learning without essentially following the existing guidelines. The future reward may be calculated using $U^{\gamma }\left(E\right)$, as definite in Equation (3), where k is the discount weight between the current and future rewards, $K\left(E,\;E^{+},\;C\right)$ is the reward assessed with the transition among states, and $Z_{EE^{+}}\left(C\right)$ is the probability of state transition.

(2) $$U^{\gamma }\left(E\right)=\sum_{C}\gamma \left(E,\;C\right)\sum_{E^{+}}Z_{EE^{+}}\left(C\right)K\left(E,\;E^{+},\;C\right)+kU^{\gamma }\left(E^{+}\right)$$

In light of this, the value iteration mechanism is calculated using Equation (4), where $U^{\gamma }\left(E^{+}\right)$ denotes the assessed value of K at $E^{+}$ in its initial repetition t and $U_{t+1}^{\gamma }\left(E^{+}\right)$ denotes the assessed value of K at the efficient iteration t+1. It is important to remember that $H\left(\left|C\right|{\left|E\right|}^{2}\right)$ it can be used for each iteration and the number of repetitions in reinforcement learning can increase exponentially.

(3) $$U_{t+1}^{\gamma }\left(E^{+}\right)=\max_{C}\sum_{E^{+}}Z_{EE^{+}}\left(C\right)K\left(E,\;E^{+},\;C\right)+kU_{t}^{\gamma }\left(E^{+}\right)$$

During training, the Q-learning utilizes a reward created by the environment for performing the learning process. The reward determines the agent’s ability as per the provided task. Rather than a prediction, the reward function helps the agent to attain a conclusion. The traditional reinforcement learning algorithm struggles to address complex issues due to (i) the sparse nature of Q-learning handling large amounts of data and (ii) the need to maintain two constraints: (a) future states and (b) future rewards for calculating the best course of action in the future. Neural networks can forecast the ideal future state and reward values for policies in order to get around these limitations. Thus, the proposed work integrates MLP with the traditional Q-reinforcement learning model. A single-hidden-layer MLP approach uses a gradient descent loss function to forecast the optimal Q-value.

(4) $$Loss\;\left(Q\left(E^{\prime },\;C^{\prime }\right)\right)=1/n\sum \left|A_{j}-Q\left(E,\;C\right)\right|$$

In this case, $A_{j}$ represents the agent (autonomous vehicle) [present state, present action, reward, new state] and $Q\left(E,\;C\right)$ represents the Q-value from the previous iteration. The ideal Q-value for the next action is anticipated using gradient descent optimization with a mean square error loss function. By utilizing MLP, continuous data and multiple constraints are effectively handled by the proposed work. Also, compared with the conventional methods, the combination of MLP can easily converge to the best Q-value. Since dynamic robot navigation in a small indoor space necessitates sequential decision-making, it can be efficiently formulated as an MDP. In order to provide dense incentives without needing the robot to rigidly obey the global direction, a unique reward function is developed. In this way, the robot promotes the merging of learning-based triangulation while inspiring the exploration of all possible options. More precisely, the reward function provides the following at each step: When the robot encounters a static or dynamic obstacle, where $k_{1}<k_{2}<0$, it receives a large negative reward ($k_{1}+k_{2}$); when it reaches a free cell that is not on the global guidance path, it obtains a small negative incentive ($k_{1}<0$); and when it reaches one of the cells on the global guidance path, it receives a large positive reward ($k_{1}+M_{e}+k_{3}$), where $k_{3}>\left|k_{1}\right|>0$ and from the moment where it initially deviates from the global guidance path until it rejoins it, $M_{e}$ is the number of cells that remain impassive. Formally, the reward function is definite as follows:

(5) $$K\left(j\right)=\left\{\begin{array}{ll} k_{1} & if\;b_{k}\left(j+1\right)\in B_{f}\backslash \gamma^{*}\\ k_{1}+k_{2} & if\;b_{k}\left(j+1\right)\in B_{s}\cup B_{d}\left(j+1\right)\\ k_{1}+M_{e}\times k_{3} & if\;b_{k}\left(j+1\right)\in \gamma^{*}\backslash B_{d}\left(j+1\right) \end{array}\right.$$

where $K\left(j\right)$ is the reward value of the action $a_{j}$ at time j, and $b_{k}\left(j+1\right)$ is the robot’s position following the action at time j. Due to its many uncertainties and both discrete and continuous high-dimensional action spaces, the MDP model is challenging for conventional RL algorithms to handle. This research proposes a novel PQLF method to address this problem. Dynamic barriers and confinement add complexity that conventional RL algorithms might not be able to handle. Because it employs function approximation to identify patterns in the data, the perceptron-Q learning fusion model adapts well to these kinds of situations and can operate in more complex or expansive contexts.

**Figure 2:**
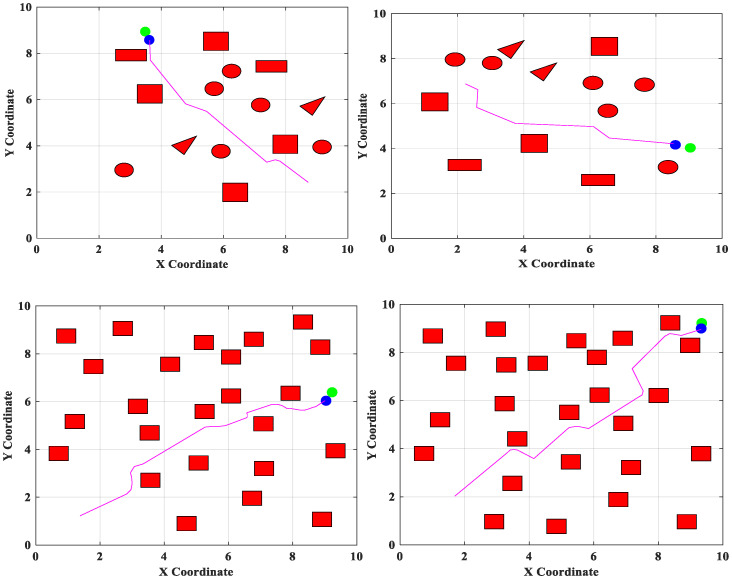
Obstacle avoidance of the proposed robot navigation system in different scenarios.

The MLP method involves an input layer, one or more hidden layers, and an output layer. Activation functions can be used to mathematically explain a complex non-linear relationship between a set of input and output variables. Weights and non-linear transfer/activation functions connect all nodes in the top and bottom layers. A node in the following tier can receive input by forwarding and scaling the output of nodes in the previous layer. The MLP outperforms other strategies for learning and expressing non-linear relations due to its ability to superimpose non-linear transfer functions. Therefore, the main advantage of MLP is its capacity to quickly resolve complicated problems. MLP has the benefit of being able to handle continuous data and numerous constraints. The inability of the perceptron to identify linear, indivisible inputs is addressed by the MLP generalization of the perceptron. Furthermore, non-linear relationships are better expressed and learned via MLP. So, compared to conventional approaches, the convergence for the optimal Q-value is quicker. The hyperparameters utilized while training the proposed PQLF model are maximum epochs-1000, optimizer-gradient descent, learning rate-0.01, $\left(\varepsilon \right)$-0.01, batch size-64, and discount factor-0.9. Figure 2 shows the obstacle avoidance and effective path-planning of the proposed robot navigation system.

**Figure 3:**
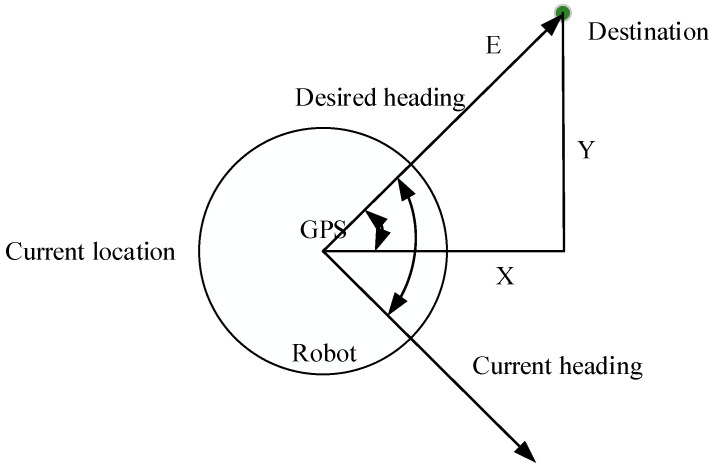
Geometric representation of robot movement.

The GPS module that is installed gives the robot its current location. Now, the heading sensor and two vector systems are used to determine the robot’s direction. Furthermore, the robot’s geometrical characteristics, GPS, and heading sensor for global path preparation are shown in Figure 3. Put the robot’s coordinates (x1 and y1) and the destination coordinates (x2 and y2) in this scenario. Equations (6)–(8) may be used to obtain the values of x and y by subtracting the robot coordinates from the final coordinates.

(6) $$E=\sqrt{X^{2}+Y^{2}}$$

(7) $$E=\sqrt{{\left(X_{2}-X_{1}\right)}^{2}+{\left(Y_{2}-Y_{1}\right)}^{2}}$$

(8) $$\phi =\tan^{-1}\left[\left(Y_{2}-Y_{1}\right)/\left(X_{2}-X_{1}\right)\right]$$

Additionally, a heading sensor is required to determine the robot’s current direction. The two-point’s vector angle computation can be used to modify the goal angle (φ). Lastly, the robot indicates that the target location has been reached if it meets the $E<E^{th}$ criterion. Algorithm 1 shows the flow of the proposed PQLF model in the robot navigation process.
**Algorithm 1:** Process of the proposed PQLF model in robot navigation Start 
Input: Action: Turn right, turn left, stop, move forward
                  State: $Z\left\{1,\ldots \ldots ,M_{s}\right\}$
Output: Best state and action
Initialize the parameters like epochs, learning rate, reward, random action, random state iteration, and mini-batch
For i to epochs do 
         For j to iteration do                 Begin in state $E_{j}\;\in \;E$
                While $E_{j}$ is not terminal do
                      Evaluate policy Q(A,C)                                        Define action ←$\;\mathrm{policy}\;\left(state\right)$                  **If** robot meets obstacle = Positive                                          reward ←$R\left(state,\;action\right)$                **else**                                          reward ←$R\left(state,\;action\right)$                                          update $\left(reward\right)$                **End if**                                          Obtained new state             For ll to mini batch **do**                                 Determine $(E_{j},C$) through MLP
$Q\left(E^{\prime },\;C\right)$ = append $\left(E^{\prime },\;C^{\prime }\right)$              End for 
            End for
End for

### 3.3. Creation of the Dataset

The dataset used to train the proposed model considers the sensor inputs and output steering angles. The dataset comprises 10,000 samples based on the sensor measurements. In Algorithm 1, the dataset was constructed using M data distances. $\phi \left(1\right)$ Continuous complexity holds true for conditional checks as well as for other operations. The key complexity is contingent on the number of distance samples Distancesample. Consequently, Algorithm 1’s average runtime is 0.0153752 s, and its worst-case time complexity is $\phi \left(Dis\tan cesample\right)$.

**Figure 4:**
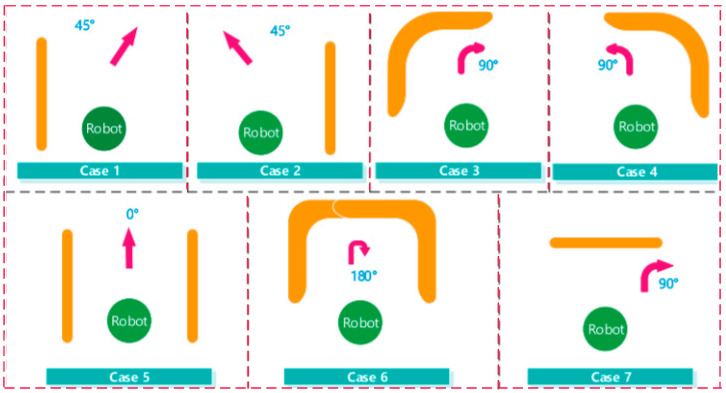
Different examples for avoiding obstructions in a congested environment.

Several of the steering control-related occurrences are clarified. As shown in Figure 4, the direction of the wheeled mobile robot is driven by seven conditions. In the first two, the robot encounters an impediment on either its left or right side, and in order to avoid it, it chooses a turning angle of ±45 degrees to the left or right. Likewise, in the third and fourth scenarios depicted in Figure 4, a ±90 degree turning angle is chosen for both the side-facing and front-facing obstacles. Additionally, the fifth instance chooses a steering angle of 0% and depicts a corridor scenario, meaning there are obstacles on both sides. To prevent the front and side-facing obstacles, a directing angle of 180% is used in the sixth scenario. 90% is the chosen steering angle to avoid the front-facing impediment. Table 1 provides further information on the steering angles and obstacle orientation, with the negative sign denoting anti-clockwise turning. In light of these situations, Algorithm 1 creates a dataset. Random variables with values between 0 and 100 are produced by Algorithm 1 in accordance with the sample ultrasonic sensor data. All of the sensors’ data is produced to train and evaluate the suggested PQLF controller. This information serves as the PQLF’s input. As stated in Table 1, the FIS output is one of the pre-defined steering controls that must adhere to the standards. Algorithm 2 is used to generate the dataset’s test and training samples in compliance with the instructions. The first column displays the data from the dataset’s left, right, and front sensors. The last column simultaneously displays the output directing angle. The generated dataset is utilized to train the proposed PQLF model.
**Algorithm 2:** Generation of datasetThreshold distance = 20 cm Distance sample = 10,000
Threshold distance = $T_{D}$
C[Distance sample, 4] = 0
t=1
                   **While**
t≤Distance sample  **do**                                      C[t, 1] = rand [100]% Left sensor data                                      C[t, 2] = rand [100]% Front sensor data                           C[t, 3] = rand [100]% Right sensor data                                       **If**
$\left(C\left[t,\;1\right]\leq T_{D}\right)\&\left(C\left[t,\;2\right]\leq T_{D}\right)\&\left(C\left[t,\;3\right]\leq T_{D}\right)$ **do**                                                           $C\left[t,\;4\right]=180$                                         **Else if**
$\left(C\left[t,\;1\right]\leq T_{D}\right)\&\left(C\left[t,\;2\right]\geq T_{D}\right)\&\left(C\left[t,\;3\right]\geq T_{D}\right)$ **then**                                             $C\left[t,\;4\right]=45$                                         **Else if**
$\left(C\left[t,\;1\right]\leq T_{D}\right)\&\left(C\left[t,\;2\right]\geq T_{D}\right)\&\left(C\left[t,\;3\right]\leq T_{D}\right)$ **then**                                                             $C\left[t,\;4\right]=0$                                         **Else if**
$\left(C\left[t,\;1\right]\leq T_{D}\right)\&\left(C\left[t,\;2\right]\geq T_{D}\right)\&\left(C\left[t,\;3\right]\geq T_{D}\right)$ **then**                                                $C\left[t,\;4\right]=90$                                         **Else if**
$\left(C\left[t,\;1\right]\geq T_{D}\right)\&\left(C\left[t,\;2\right]\leq T_{D}\right)\&\left(C\left[t,\;3\right]\leq T_{D}\right)$ **then**                                                $C\left[t,\;4\right]=90\%\;\left(Anti\;clockwise\right)$                                         **Else if**
$\left(C\left[t,\;1\right]\geq T_{D}\right)\&\left(C\left[t,\;2\right]\geq T_{D}\right)\&\left(C\left[t,\;3\right]\leq T_{D}\right)$ **then**                                                $C\left[t,\;4\right]=45\%\;\left(Anti\;clockwise\right)$                                         **Else if**
$\left(C\left[t,\;1\right]\geq T_{D}\right)\&\left(C\left[t,\;2\right]\leq T_{D}\right)\&\left(C\left[t,\;3\right]\geq T_{D}\right)$ **then**                                                $C\left[t,\;4\right]=90$                           **End if** t=t+1 **End while**
**End**

## 4. Results and Discussion

The 3D robotics replication platform CoppeliaSim, created by Coppelia Robotics and formerly recognized as V-REP, is an integrated development environment that enables the modeling, editing, programming, and simulation of a robot utilizing sensors. It offers a wide range of features that are simple to combine with a comprehensive API and scripting capabilities. CoppeliaSim is integrated with MATLAB to validate the suggested methodology in this study. MATLAB 2021a and CoppeliaSim 4.2.0 are used for all training and testing. A wheeled mobile robot called “Pioneer 3-DX” with several obstacles and a resolution point is incorporated into the simulation scenarios for the experiment. To balance the robot’s construction, the Pioneer 3-DX robot incorporates a passive omni wheel at the back and two separate motor-controlled front wheels. In every simulation, the mobile robot moves at a speed of 0.2 m/s. 10,000 random samples of left, right, and front sensor reserves are used to create the dataset using Algorithm 1. Furthermore, the input sensor data is given a label (steering angle) depending on the threshold. In trials, PQLF is tested using 25% of the dataset, and PQLF is trained using 75% of the dataset. All simulation experiments and datasets used for training and testing the proposed PQLF model were generated internally in CoppeliaSim and are not based on any external datasets. For comparison with prior approaches, performance metrics (moving cost, detour percentage, computation time, and success rate) were adopted. These are widely used in dynamic robot navigation studies. To ensure compatibility and fair adaptation, each metric was implemented in the proposed framework following the definitions in [25]. Moving cost was computed as the ratio between actual path length and the Manhattan distance. Detour percentage was calculated as the excess path length relative to the shortest path. Computation time was measured as the average per-step processing time in seconds. The success rate was determined as the percentage of trials in which the robot reached the goal without collision. These definitions were carefully validated within the proposed simulator to match the formulations reported in the baseline study. No external raw data were reused in this work; only the metric definitions were adapted to ensure direct comparability with prior methods.

### 4.1. Performance Matrix

The performance evaluation matrix is as follows:

#### 4.1.1. Detour Percentage [26]:


(9) $$Detour\;percenage\;=\;\frac{S_{n}-A_{L}\left(D_{goal}\;D_{start}\right)}{A_{L}\left(D_{goal}\;D_{start}\right)}\times 100\%$$

The length of the short path between the goal and the initial position is represented as $A_{L}\left(D_{goal}D_{start}\right)$, which is estimated as $L^{*}$ algorithm by considering static obstacles. With respective to the path with the shortest length, the metric specifies the detour percentage.

#### 4.1.2. Moving Cost:

This statistic compares real movement steps to the desired number of steps without barriers [27].

(10) $$Movingcost=\frac{S_{n}}{\left\|D_{start}-D_{goal}\right\|M_{1}}$$

where $\left\|D_{start}-D_{goal}\right\|$$\;M_{1}$ represents the Manhattan distance from the start to the destination. The number of steps taken is represented in $S_{n}$.

#### 4.1.3. Computation Time:

This statistic represents the average computation time at each phase of the testing.

**Figure 5:**
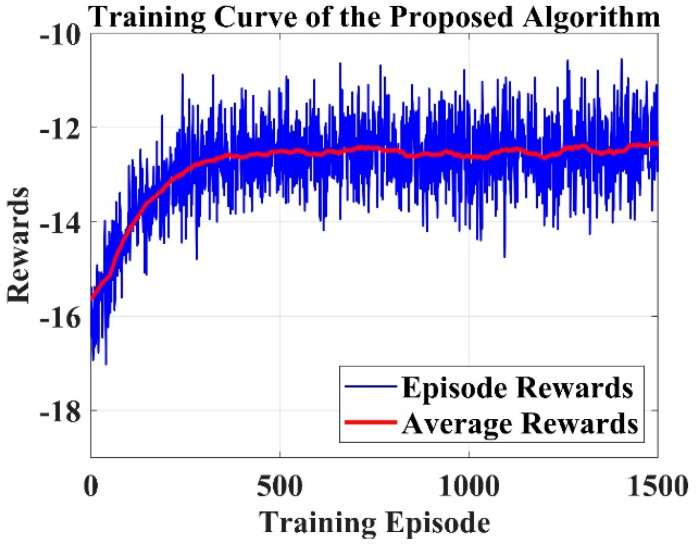
Reward vs. training episodes.

**Figure 6:**
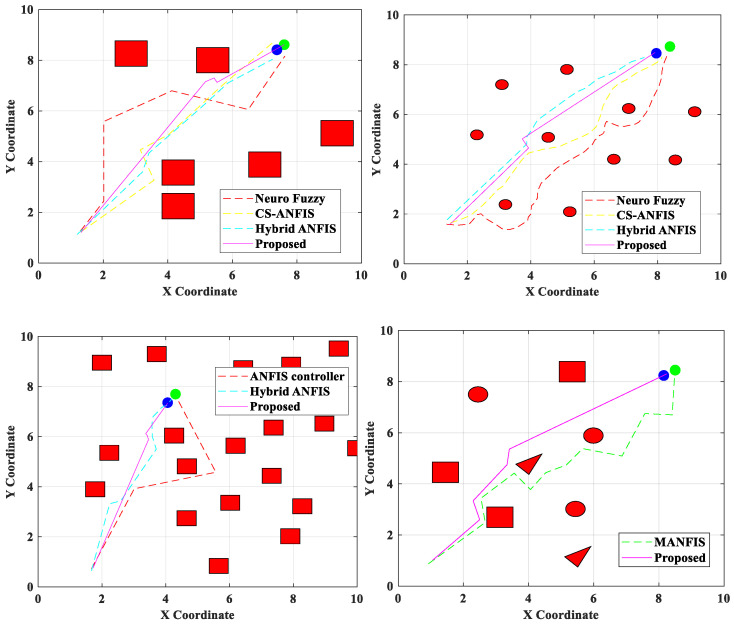
Comparison of the proposed model with existing models in various environments.

Figure 5 illustrates the reward versus training episodes for the proposed PQLF model. The results were obtained in the Regular100 environment configuration, which provides a balanced obstacle density for evaluating navigation stability. Each curve represents the average performance over 10 independent training runs, with randomized initial positions and obstacle layouts to ensure robustness. The blue curve corresponds to the raw episode rewards, while the red curve shows the smoothed average rewards, reflecting the model’s progressive improvement in decision-making. The upward trend indicates that the PQLF agent gradually learns to maximize cumulative rewards by consistently avoiding collisions and reaching the goal. The performance of a dynamic robot navigation system trained with a PQLF in a small interior space is shown in Figure 5. The red curve displays the smoothed average rewards, while the blue curve depicts the prizes earned throughout each training session. The model’s learning progress as it adjusts to the environment and gradually improves its navigation choices is seen by the rising trend in average rewards. Reflecting the complexity of the environment through the variation in episode rewards, the model becomes more resilient and efficient as training progresses. The efficiency of PQLF in managing dynamic impediments and high-dimensional action environments is demonstrated. Figure 6 shows the comparison analysis of proposed and existing models in various environments.

**Figure 7:**
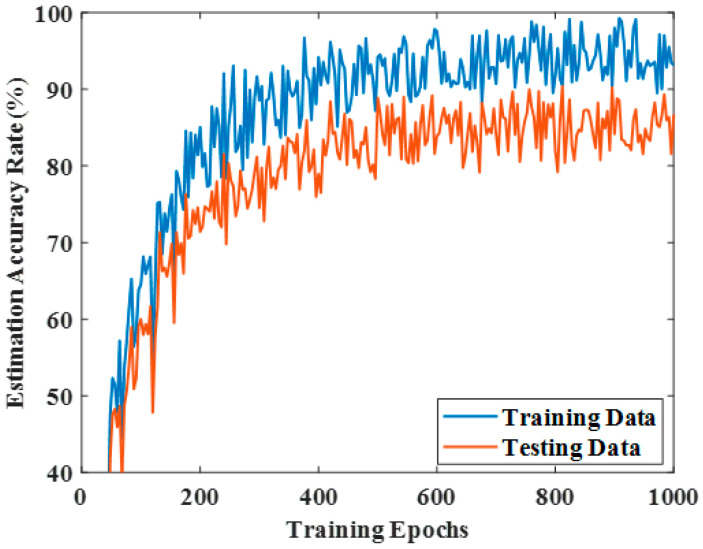
Accuracy rate analysis.

Figure 6 presents a comparative visualization of the proposed PQLF model against several baseline approaches, namely Local re-planning, Global re-planning, Naïve re-planning, and G2RL [25]. These are the same baselines reported numerically in Table 2, Table 3 and Table 4; the figure provides a graphical summary to highlight performance differences across methods. No additional or unexplained models beyond these baselines are included. The environments used for Figure 6 are the Regular, Random, and Free configurations described earlier, each tested at obstacle counts of 50, 100, and 150. To avoid ambiguity, the figure caption has been revised to explicitly state both the baseline models included and the environment settings under which the comparison was performed. This ensures consistency between the figure and the corresponding tables, while also clarifying that the purpose of Figure 6 is to provide a trend-level visualization rather than introduce new models. For further evaluation of the performance of the proposed PQLF model, the accuracy rate is determined with respect to the training epochs. To validate the strength of the proposed model, the epochs are varied from 0 to 1000. By varying the epochs, the accuracy of the proposed model is enhanced in the obstacle avoidance task. Figure 7 shows the training performance analysis through the accuracy rate estimation.

**Figure 8:**
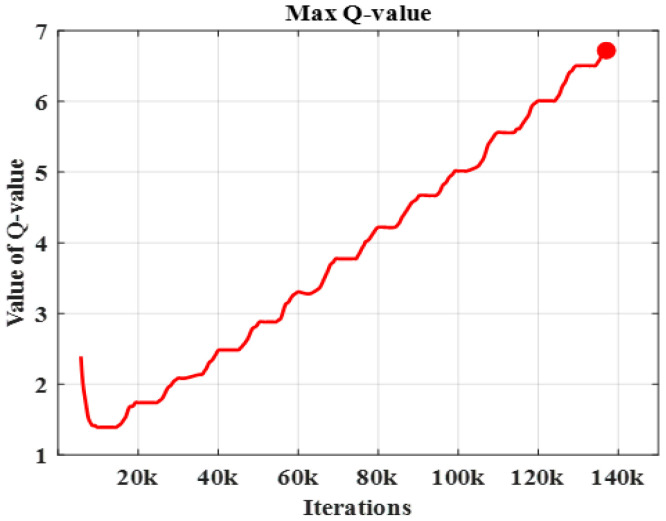
Maximum Q-value.

The analysis of the accuracy rate clearly exhibits the efficiency of the proposed work. This shows that the proposed PQLF model is highly sufficient for the robot navigation system. Figure 8 represents the maximum Q-value attained in the proposed model.

From Figure 8, it is observed that the proposed model obtains an increased Q-value in each iteration to attain the maximum benefits. Through this result analysis, the strength of the proposed model is revealed.

### 4.2. Performance Comparison Using Various Models

For each environment type (Regular, Random, and Free), the experiments were repeated over 30 independent trials per configuration (e.g., Regular50, Random100, Free150). In each trial, the robot was initialized at a random starting position and oriented towards a predefined goal location, with obstacle placement.

The success percentage [28] reported was computed as

(11) $$Successrate(\%)=\frac{Numberof\;successfultrails}{Totaltrials}\times 100$$

A trial was considered successful if the robot reached the target without collision within the maximum step limit. For quantitative metrics such as moving cost, detour percentage, and computation time (Table 2, Table 3 and Table 4), the values represent the mean performance across the 30 trials. Standard deviations were also calculated to assess variability, and results were verified using a two-sample *t*-test to ensure that the improvements achieved by the proposed PQLF model over baselines were statistically significant at the *p* < 0.05 level. This evaluation protocol ensures that the reported performance is not due to chance but reflects the consistent effectiveness and robustness of the proposed navigation model across different obstacle densities and environment configurations.

To maintain clarity across evaluations, the set of baseline methods has been standardized. Specifically, Table 2, Table 3 and Table 4 (moving cost, detour percentage, and computation time) report comparisons with Local re-planning, Global re-planning, Naïve re-planning, and G2RL [25], as these provide directly compatible metrics. Figure 11 (computation time) has been aligned with the same baselines to ensure consistency. Table 5 (success rate) includes these four baselines together with PRIMAL and Discrete-ORCA [25], since success rate results for these methods are available in prior literature. Finally, Table 6 and Figure 14 (reward analysis) extend the comparison to additional DRL-based methods (DQN, DDPG, PPO, and D-PDQN) in order to assess training efficiency and policy stability. This standardized framework ensures that each metric is evaluated against the most relevant baselines, while also providing broader coverage where results from recent DRL models are directly comparable.

**Figure 9:**
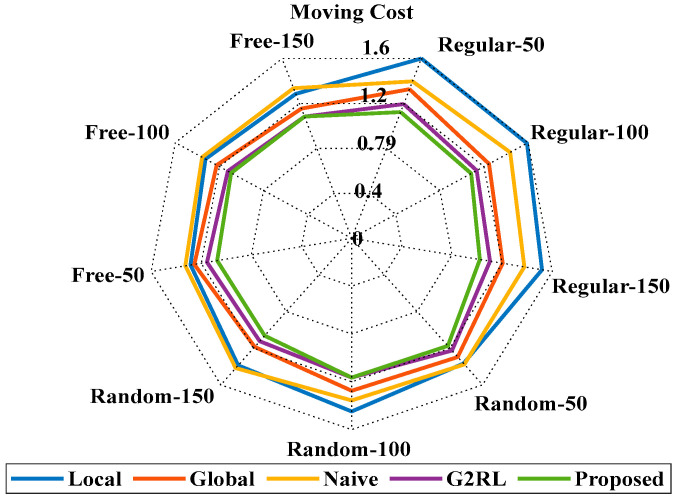
Moving cost of various models.

Figure 9 represents the moving cost [25] comparison of proposed and existing models in regular, random, and free conditions. Models like local re-planning, global and naïve re-planning, and G2RL are used to compare with the proposed model. By comparing various models, the proposed model attained the average best value of 1.0844. So, the proposed model attained the smallest moving steps among all other existing models. The detailed values are presented in Table 2.

To ensure a fair evaluation of the proposed PQLF model, three distinct types of testing environments were designed in the CoppeliaSim simulator, each reflecting different levels of obstacle density and placement. Regular environment (Regular50/100/150): A structured indoor map with evenly spaced static obstacles such as walls and furniture. The suffix (50, 100, 150) indicates the number of obstacle elements present in the environment. Random environment (Random50/100/150): An environment where obstacles are placed at random positions and orientations within the confined space, introducing unpredictability in navigation. Again, the suffix denotes the number of obstacles. Free environment (Free50/100/150): A relatively open configuration with sparse obstacles, providing wide corridors or free space for navigation. The suffix indicates the number of obstacle elements considered, though these are sparsely distributed compared to the regular and random cases. These environments were systematically generated in CoppeliaSim using randomized obstacle placement scripts (for Random), grid-based placement templates (for Regular), and low-density spatial arrangements (for Free). Each environment type was tested with different obstacle counts (50, 100, and 150) to analyze the scalability and robustness of the navigation model.

**Figure 10:**
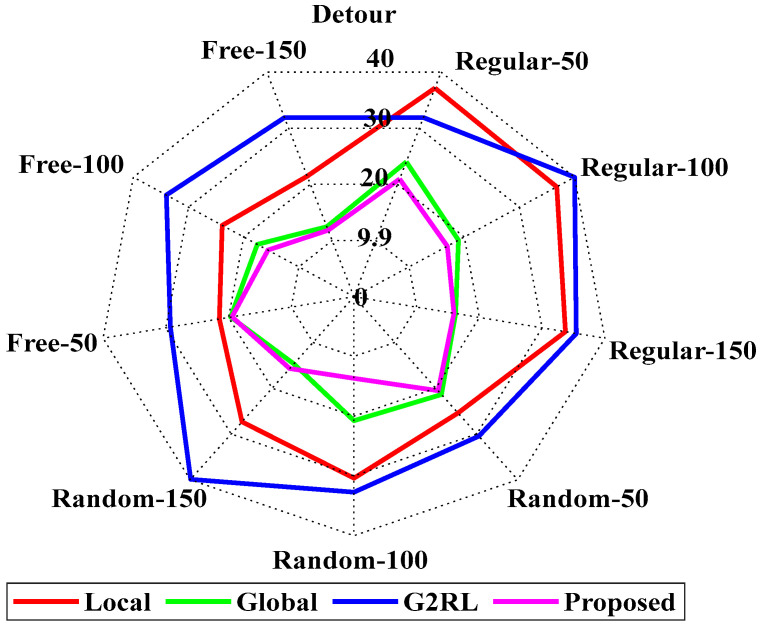
Detour percentage analysis of various models.

Figure 10 represents the detour percentage [25] of the suggested and prevailing models. The suggested model attained a value of 15.755 at the regular 150 and a value of 15.5 at the random 150, respectively. The proposed model attained the value of 11.7 at the free 150 with an overall average detour percentage of 16.528%. The suggested model attained the best results in each condition. The detailed values are presented in Table 3.

In the proposed framework, it is important to distinguish between training episodes and training epochs. A training episode corresponds to one complete navigation trial in the reinforcement learning loop, where the robot starts from an initial state and interacts with the environment until a terminal condition (goal reached or collision) is satisfied. At the end of each episode, the cumulative reward is calculated and used to update the policy. Thus, Figure 5 reports the evolution of the reward function across multiple episodes, reflecting the policy improvement in the reinforcement agent over time. On the other hand, a training epoch denotes a complete pass through the supervised dataset generated from sensor inputs and steering control outputs (10,000 samples). The perceptron module within the PQLF model is optimized using gradient descent across these epochs. Figure 7; therefore, illustrates the accuracy performance of the PQLF during supervised learning of sensor-to-action mappings. The apparent difference between the values in Figure 5 and Figure 7 arises because episodes measure the agent–environment interactions in reinforcement learning, whereas epochs measure the convergence behavior of the perceptron training in supervised learning. The reward function used in this study is structured as follows,

(12) $$R\left(s_{t},a_{t}\right)=\left\{\begin{array}{ll} -R_{obs} & if\;the\;robots\;collides\;with\;an\;obstacle\\ -R_{dev} & if\;the\;robots\;deviates\;from\;the\;goal\;path\\ +R_{goal} & if\;the\;robots\;sucessfully\;reaches\;the\;goal \end{array}\right.$$

where $R_{obs}$ is a large negative reward, $R_{dev}$ is a small negative reward, and $R_{goal}$ is a large positive reward. This design provides dense feedback to the agent, encouraging goal-directed behavior while effectively penalizing collisions and unnecessary deviations.

**Figure 11:**
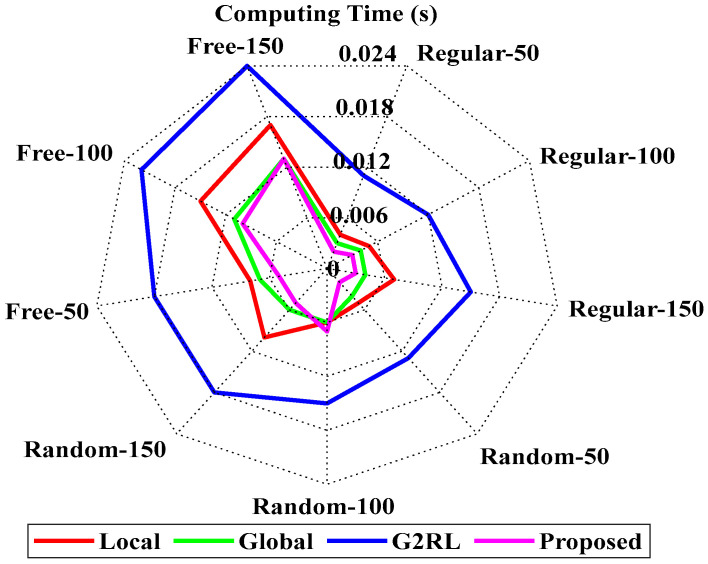
Analysis of computational time.

Figure 11 represents the computing time analysis of the proposed and existing models [25]. The suggested model attained the value of 0.002 at the regular 50, which slightly increases to 0.003 at the regular 150. The proposed model attained the values of 0.005 and 0.013 at random 150 and free 150, respectively. The results state that the proposed model has the best computing time among all other existing models. The detailed computational time ranges of the proposed and existing methods are presented in Table 4. Figures and tables in this study are intended to complement each other rather than duplicate information. Specifically, Table 4 provides the exact numerical values of computation time for each baseline method across all environments, whereas Figure 11 offers a visual comparison to highlight overall performance trends and relative differences between models. While the table enables precise quantitative reference, the figure allows readers to more easily interpret scalability and relative efficiency across environments. A similar rationale applies to other figure–table pairs, where tables report detailed values and figures present summarized trends for clearer interpretation.

**Figure 12:**
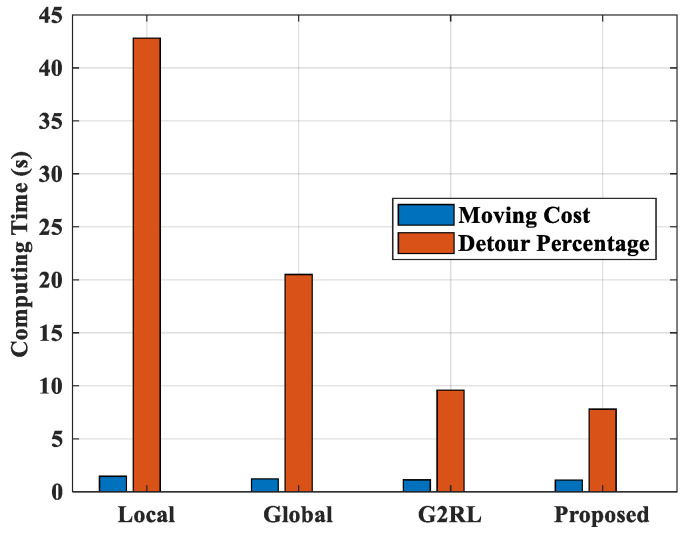
Performance evaluation in an unseen environment.

Figure 12 represents the moving cost and detour percentage of proposed and existing models in an unseen environment. The Local re-planning models attained a moving cost of 1.46 and a detour percentage of 42.8%. The Global and G2RL models have moving costs of 1.21 and 1.12, with detour percentages of 20.5% and 9.6%. The proposed model attained the best values of 1.1 for moving cost and 7.8% for detour percentage. The results show the superiority of the suggested model over all other prevailing models.

**Figure 13:**
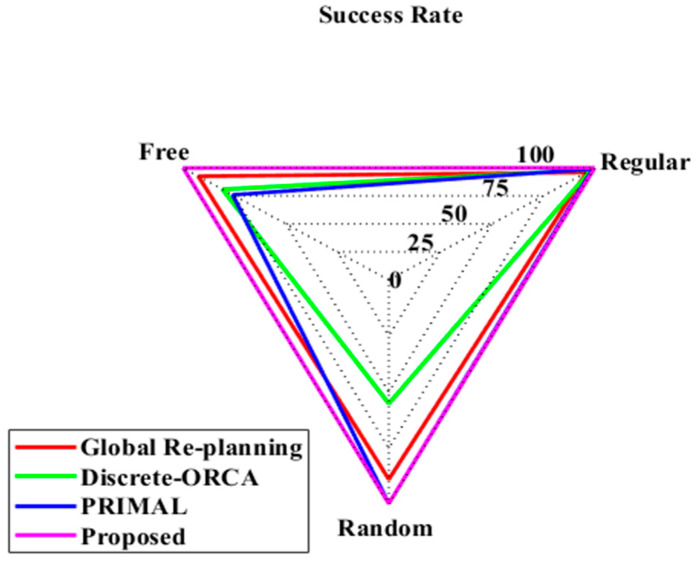
Success rate analysis of proposed and existing models.

Figure 13 represents the success rate of the suggested and prevailing models. The global re-planning model has a success rate of 95.7% at regular and attained values of 98.2% and 98.2% in free and random environments. The discrete ORCA model attained values of 88.7%, 55.0%, and 99.5% in regular, random, and free environments, respectively. The Primal model attained the value of 92.3%, 80.6% and 99.8% in regular, random, and free environments. The proposed model attained the best results of 99.8%, 99.5% and 99.6% at regular, random, and free environments, respectively. The success rate values of various models are given in Table 5.

The baseline results for Local re-planning, Global re-planning, Naïve re-planning, and G2RL models were taken from Wang et al. [25], which introduced the globally guided reinforcement learning framework. While the experimental environments proposed study (Regular, Random, and Free with 50/100/150 obstacles) were implemented in CoppeliaSim, the corresponding obstacle densities and map scales were aligned with the descriptions in [24] to ensure as much consistency as possible. The robot kinematics (three-wheeled differential drive model), sensor modalities, and speed were also matched to the specifications reported in [25]. Nevertheless, that absolute comparability cannot be guaranteed due to differences in simulator implementation (CoppeliaSim vs. original testbed) and minor variations in parameter tuning. To mitigate this, the proposed study followed the same evaluation metrics (moving cost, detour percentage, computation time, and success rate) as defined in [25], and ensured consistent definitions of reward and termination conditions. The reported improvements, therefore, reflect the relative robustness of the proposed PQLF approach under equivalent evaluation settings, even if the environments are not strictly identical to those in the baseline paper.

**Figure 14:**
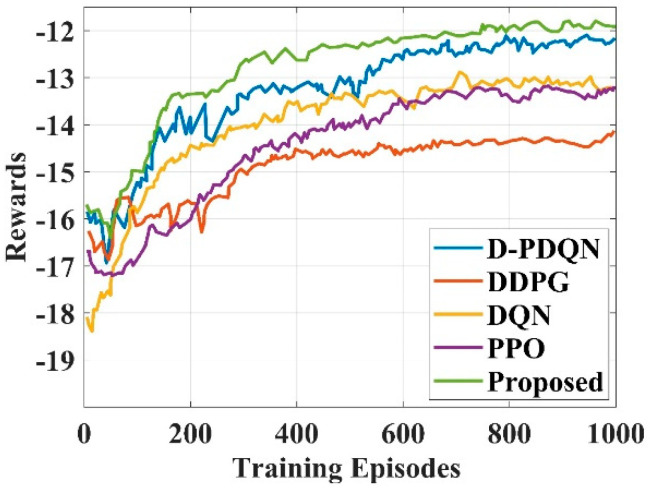
Rewards vs. training episodes.

In this study, the primary comparative focus was on methods reported in Wang et al. [25] (Local re-planning, Global re-planning, Naïve re-planning, and G2RL), since these approaches define widely accepted benchmarks in dynamic robot navigation under similar evaluation settings. These were selected to ensure consistency of metrics (moving cost, detour percentage, computation time, success rate) and to allow direct comparison with the state-of-the-art framework most relevant proposed study. Other navigation approaches, such as DQN-based policies, A* combined with DWA, or SLAM-integrated planners, were not included in Table 2, Table 3, Table 4 and Table 5 because their performance results were not directly reported in [25] under comparable experimental conditions. However, to strengthen the scope of evaluation, a broader set of baselines (DQN, DDPG, PPO, D-PDQN) was included in the reward analysis (Table 6, Figure 14) and also compared energy efficiency (Table 7 and Table 8), thereby ensuring that the proposed PQLF was assessed against both classical planning-based and modern reinforcement learning-based strategies. Compared with recent vision-based navigation approaches such as ViNT [11], which leverage large-scale visual foundation models for robust generalization, the proposed PQLF framework adopts a sensor-driven strategy using ultrasonic distance measurements. While vision-based methods offer richer scene understanding and adaptability in unstructured environments, they typically require high computational resources, large training datasets, and powerful hardware for real-time processing. In contrast, the PQLF model is lightweight, relies on simpler sensor inputs, and achieves efficient obstacle avoidance and path planning with significantly reduced computation time (0.003–0.013 s per step). This trade-off highlights that vision-based methods are more suitable for complex semantic reasoning tasks, whereas the PQLF approach is advantageous for real-time deployment in resource-constrained mobile robots.

#### Statistical Analysis

To validate the robustness of the reported results and avoid the possibility of overfitting or cherry-picked scenarios, a detailed statistical analysis was performed. Each configuration (Regular, Random, Free at obstacle counts of 50, 100, and 150) was tested with 30 independent trials. For each trial, the robot’s starting position and orientation were randomized, and in the Random setting, obstacle placements were also regenerated. The success rate values reported earlier represent the mean across these trials. In addition, the standard deviation (SD) and 95% confidence intervals (CIs) were computed to assess variability. Table 6 presents the statistical robustness of the proposed PQLF model compared to the strongest baseline (G2RL [25]) in selected configurations.

The results confirm that the proposed PQLF consistently outperforms the baseline, with improvements that are statistically significant at the 95% confidence level (*p* < 0.05, two-sample *t*-test). The low standard deviations (<±1.2%) indicate stable performance across repeated trials, while the narrow confidence intervals demonstrate reliability in success rate estimation. This analysis provides strong evidence that the high success rates reported in earlier tables are not isolated cases but reflect a robust and repeatable performance of the PQLF model in dynamic and constrained environments.

### 4.3. Discussion

A novel PQLF model is introduced for Robot Navigation in this study, while a generic three-wheeled robot model with two front wheels and a passive omni wheel in the back will be employed. Three sensors are positioned around the robot’s left, right, and front sides, respectively, and a GPS module is positioned in the middle of the device. The global navigation controller receives data from GPS. Likewise, the perceptron-Q learning fusion model is fed by ultrasonic sensors. Before deciding which way to move, the robot must first determine whether or not there are any obstacles below the threshold. During local path-planning, the sensors provide the robot with information about the distances of close, mid, and far obstacles.

The PQLF Model-based navigation controller of the robot will receive these details as input and function as an agent. It uses the current state to determine the robot’s behaviors (such as moving forward, turning left, turning right, or stopping). Goal locations (goal places for navigation) and a constrained indoor area with barriers (such as walls and furniture) make up the environment. The environment provides feedback in the form of obstacle distances (near, mid, far) from ultrasonic sensors and GPS data for the robot’s global position. And collision detection if the robot makes an invalid move. The state is defined by sensor readings, the robot’s position and location, and the Coordinates or region of the target location. Possible actions in this problem are moving forward, turning left, turning right, and stopping. Also, a new reward function is defined to provide a large negative reward when the robot encounters an obstacle, a small negative reward when it deviates from the goal, and a large positive reward when the goal is attained. The agent aimed to make the most of the cumulative predictable reward, which is the sum of all rewards over time. Thus, it is possible to formulate the Dynamic Robot Navigation in a Confined Indoor Environment as an MDP. Figure 14 represents the reward function [25] of various models by varying training episodes.

The comparison of the five DRL approaches in terms of policy quality and training speed is displayed in Figure 14. All algorithms go through a gradual learning phase in the beginning, with the goal of investigating the surroundings and gathering experience. The cumulative gains of all approaches rise quickly and then level off as learning becomes more profound. The proposed model outperforms DQN, DDPG, and D-PDQN in terms of learning time and policy quality. This is mostly because the actor and critic mine mixed action space for more information, and the double Q-value improves stability. The extension of discrete spaces to continuous spaces in DDPG surely expands the search space and decreases efficiency. The DQN loses a lot of important action information due to the rough split of its continuous action space. Furthermore, because the PPO employs an on-policy mechanism, every batch of used data is immediately destroyed, which results in a very low data utilization rate and subpar policy performance. The proposed model attained a value of 4.305 at 0 training episodes and increased to 6.649 at 200 training episodes. The proposed model attained values of 7.373, 7.844, and 8.052 at the training episode of 400, 600, and 800, respectively. The suggested model attained a value of 8.088 at the training episode of 1000. Table 8 represents the reward analysis of the suggested and prevailing models.

**Figure 15:**
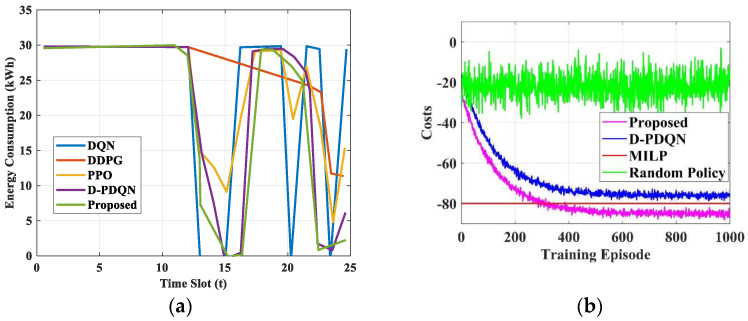
(**a**) Energy consumption analysis (**b**) Cost comparison analysis.

The results show the superiority of the suggested model by associating it with other prevailing models. Figure 15 represents the energy consumption and cost of various models. The proposed model attained an energy consumption [25] of 17.16 kWh and has a cost of −71.78, correspondingly. The suggested model attained the optimal values for these parameters. Table 9 and Table 10 represent the energy consumption and cost of the suggested and prevailing models.

The adaptability of the system arises from the hybrid architecture; the perceptron generalizes to unseen sensor states through function approximation, while Q-learning ensures continuous policy improvement by interacting with the environment. This combination enables the robot to adjust effectively to new and dynamic obstacle configurations. Second, the runtime efficiency is explained by the lightweight design of the model. The single-hidden-layer perceptron requires only linear-time forward propagation, and Q-learning updates are tabular and computationally inexpensive. Together, these components significantly reduce the computational burden compared to deep reinforcement learning approaches. This theoretical efficiency is consistent with the observed per-step computation time of 0.003–0.013 s, demonstrating suitability for real-time deployment on embedded robotic platforms. Finally, the stability of training is reinforced by the structured reward function, which mitigates the sparse reward problem by providing dense and balanced rewards. This design accelerates convergence and explains the steady improvement observed across training episodes.

The superior performance of the proposed PQLF model arises from its hybrid design, which combines the predictive capability of an MLP with the decision-making ability of Q-learning. The perceptron improves the estimation of Q-values by handling continuous sensor data, while Q-learning provides robust exploration and exploitation balance for discrete navigation actions. To objectively demonstrate why this integration is dominant, an ablation study and a sensitivity analysis are performed. Three variants of the proposed system were tested under identical conditions (Regular100, Random100, Free100 environments). Classical tabular Q-learning with no perceptron. A supervised MLP trained to map sensor data to steering actions without reinforcement signals, and a Full hybrid model with the designed reward function. The average results across 30 trials are shown in Table 11.

These results confirm that while both QL and MLP alone can achieve feasible navigation, the fusion (PQLF) achieves significantly better performance across all metrics. The perceptron reduces approximation error in Q-values, while Q-learning provides long-term optimal policy exploration.

**Sensitivity Analysis:**

The robustness of PQLF was further evaluated by varying three critical hyperparameters: learning rate α, discount factor γ, and exploration rate ε. Table 12 shows the impact of changing these parameters on the success rate in Regular100 environments.

The results show that performance remains consistently high across a wide range of parameters, with only slight reductions at extreme values. This indicates that the PQLF framework is not overly sensitive to hyperparameter tuning, making it practical for deployment in real-world robotic systems.

### 4.4. Limitations and Boundaries of the Proposed Approach

The proposed PQLF model demonstrates superior navigation performance across various simulated indoor environments, but also has several limitations. All experiments were conducted in CoppeliaSim with MATLAB integration. Although simulation offers controlled testing, real-world environments involve additional uncertainties such as sensor noise, actuator delays, wheel slippage, and communication losses, which may affect model robustness. Although the model achieves reduced computation time compared to baselines, training still requires large datasets (10,000 samples) and multiple episodes, which may limit scalability for multi-robot systems or real-time online learning. While several benchmarks were included, some traditional path-planning methods (e.g., SLAM-integrated A* + DWA planners) were not implemented proposed testbed, restricting the breadth of comparison. Future research will address these boundaries by validating the approach on physical robot platforms, extending experiments to outdoor and large-scale environments, investigating adaptive or learned reward functions, and benchmarking against a wider range of classical and modern navigation algorithms. Another important limitation of this study is the assumption of error-free GPS measurements. Since the experiments were performed in CoppeliaSim, the GPS module provided idealized location data without accounting for multipath effects or signal loss. In real indoor environments, GPS suffers from poor accuracy due to the absence of line of sight, which can significantly increase navigation errors. While this assumption was acceptable for validating the feasibility of the proposed PQLF model, future work will integrate more realistic indoor localization methods such as Ultra-Wideband (UWB), LiDAR-SLAM, or vision-based positioning to ensure robust performance in practical deployments.

## 5. Conclusions

In this research, a new combination of Q-reinforcement learning and MLP model is presented for Robot Navigation. This study utilizes a typical three-wheeled robot model that has a passive omni wheel in the back and two front wheels. Similarly, ultrasonic sensors feed the perceptron-Q learning fusion model. The robot uses the sensors to determine the distances of nearby, intermediate, and distant obstacles during local path-planning. These details are then sent to the robot’s PQLF Model-based navigation controller, and it acts as an agent. The cumulative anticipated reward, or the total of all rewards throughout time, is the goal the agent sought to optimize. Through the proposed PQLF, effective robot navigation is performed by adjusting the reward function. The simulation results show that the proposed model attained an energy consumption of 17.16 kWh and a reduced cost of −71.78, respectively. Also, the proposed study obtains a minimized moving cost of 1.1 and a detour percentage of 7.8%. The comparison analysis shows that the proposed model outperforms other existing robot navigation models. In the future, this research would like to include more advanced techniques to enhance performance even further and obtain the best results. Furthermore, autonomous vehicles are prone to cyber-attacks, so an advanced attack prevention system will be introduced in the future study. Additionally, this study was conducted only in an indoor environment, so in future studies, this research would like to extend it further by including outdoor environments as well.

## Figures and Tables

**Table 1:** Details of Steering control for training PQLF.

Case 1	Steering Angle°	Orientation of Obstacles
1	90	Front
2	0	Corridor
3	90	Left front corner
4	45	Left
5	−45 (anti clockwise)	Right
6	−90 (anti clockwise)	Right front corner
7	180	Left, Front, Right

**Table 2:** Moving cost comparison of proposed and existing models.

Moving Cost					
	Local	Global	Naïve	G2RL	Proposed
Regular 50	1.58	1.31	1.38	1.18	1.11
Regular 100	1.57	1.23	1.42	1.12	1.07
Regular 150	1.50	1.19	1.36	1.09	1.01
Random 50	1.35	1.28	1.36	1.21	1.16
Random 100	1.43	1.26	1.34	1.15	1.15
Random 150	1.37	1.17	1.40	1.11	1.05
Free 50	1.27	1.24	1.31	1.14	1.06
Free 100	1.31	1.21	1.34	1.11	1.08
Free 150	1.27	1.14	1.32	1.07	1.07

**Table 3:** Detour percentage comparison of proposed and existing models.

Detour Percentage (%)					
	Local	Global	Naïve	G2RL	Proposed
Regular 50	36.7	23.7	31.5	15.2	20.7
Regular 100	36.3	18.7	39.5	10.7	16.7
Regular 150	33.3	16.0	35.0	8.2	15.755
Random 50	25.1	21.1	30.1	16.7	20.3
Random 100	30.0	20.5	32.3	13.0	13.5
Random 150	27.0	14.5	39.4	9.1	15.5
Free 50	21.2	19.4	28.9	12.3	19.2
Free 100	23.6	17.3	33.6	9.1	15.4
Free 150	21.2	12.3	31.5	6.5	11.7

**Table 4:** Computational time comparison of proposed and existing methods.

Computing Time (s)				
	Local [25]	Global [25]	G2RL [25]	Proposed
Regular 50	0.004	0.003	0.011	0.002
Regular 100	0.005	0.004	0.012	0.003
Regular 150	0.007	0.004	0.015	0.003
Random 50	0.005	0.004	0.013	0.002
Random 100	0.006	0.006	0.015	0.007
Random 150	0.10	0.006	0.018	0.005
Free 50	0.008	0.007	0.018	0.005
Free 100	0.15	0.011	0.022	0.01
Free 150	0.17	0.013	0.028	0.013

**Table 5:** Success rate of proposed and existing models.

Methods	Global Re-Planning [25]	Discrete-ORCA [25]	PRIMAL [25]	Proposed
Regular	95.7%	88.7%	92.3%	99.8%
Random	98.2%	55.0%	80.6%	99.5%
Free	98.8%	99.5%	75.7%	99.6%

**Table 6:** Statistical analysis of success rates (30 trials per setting).

Environment	Model	Mean Success Rate (%)	SD (±)	95% CI (Lower–Upper)
Regular100	G2RL [25]	98.2	1.8	96.3–99.5
Regular100	Proposed	99.8	0.7	99.1–100
Random150	G2RL [25]	90.9	2.3	88.4–93.2
Random150	Proposed	99.5	0.9	98.7–100
Free150	G2RL [25]	93.5	1.9	91.6–95.4
Free150	Proposed	99.6	1.0	98.8–100

**Table 7:** Comparison of PQLF with Vision-Based Navigation [27].

Feature/Criterion	Proposed PQLF	Vision-Based Navigation
Primary Input	Ultrasonic distance sensors + GPS (global reference)	Visual input (RGB images, depth maps, semantic features)
Computation Requirement	Lightweight (0.003–0.013 s per step, suitable for embedded use)	High (requires GPUs/accelerators for real-time inference)
Training Data	Generated internally (10,000 sensor samples, synthetic dataset)	Requires large-scale labeled/unlabeled visual datasets
Generalization Ability	Strong in structured indoor environments with sensor cues	Stronger in unstructured, dynamic, and semantically rich scenes
Real-time Deployment	Highly suitable for low-power robots with limited resources	More challenging on resource-constrained platforms
Strength	Efficient obstacle avoidance, fast decision-making	Rich perception, semantic reasoning, adaptability
Limitation	Relies on simplified sensors, less context awareness	Computationally expensive, sensitive to lighting/occlusions

**Table 8:** Reward analysis of the suggested and existing models.

Algorithms	Training Episodes					
	0	200	400	600	800	1000
D-PDQN	4.153	6.161	6.776	7.391	7.744	7.844
DDPG	3.744	4.323	5.482	5.473	5.663	5.871
DQN	1.916	5.554	6.495	6.495	6.921	6.794
PPO	3.31	3.997	5.817	6.341	6.504	6.821
Proposed	4.305	6.649	7.373	7.844	8.052	8.088

**Table 9:** Energy Consumption of the proposed and existing models.

Energy Consumption (kWh)					
Time Slot (t)	5	10	15	20	25
DQN	29.804	29.715	0.692	0.54	29.426
DDPG	29.804	29.715	29.715	24.257	11.351
PPO	29.804	29.715	9.115	19.459	15.388
D-PDQN	29.804	29.715	0.499	28.304	6.157
Proposed	29.559	29.982	0.072	26.982	2.249

**Table 10:** Energy Cost analysis of proposed and existing models.

Energy Cost						
Training Episode	0	200	400	600	800	1000
Proposed	−20	−72.55	−83.11	−84.7	−85.51	−84.84
D-PDQN	−10	−63.79	−74.0167	−75.6226	−75.7899	−75.4658
MILP	−80	−80	−80	−80	−80	−80
Random Policy	−20.3932	−18.1775	−25.9067	−23.8075	−20.5935	−23.8533

**Table 11:** Ablation study of QL, MLP, and PQLF.

Model	Moving Cost	Detour (%)	Success Rate (%)	Avg. Comp. Time (s)
Q-learning	1.32	24.6	91.2	0.007
MLP only	1.25	21.7	94.3	0.006
Proposed PQLF	1.08	15.5	99.5	0.003

**Table 12:** Sensitivity analysis of PQLF (success rate %).

Parameter Varied	Value Tested	Success Rate (%)
Learning rate α	0.001/0.01/0.1	96.7/99.5/98.2
Discount factor γ	0.7/0.9/0.99	95.6/99.5/99.0
Exploration rate ε	0.1/0.01/0.001	94.8/99.5/97.4

## Data Availability

All research data are included in the article.

## Abbreviations

AV	Autonomous Vehicle
DQN	Deep Q-network
DDPG	Deep Deterministic Policy Gradient
ESS	Energy Storage Systems
GPS	Global Positioning System
MDP	Markov Decision Process
MLP	Multi-Layer Perceptron
PQLF	Perceptron-Q Learning Fusion
QL	Q-learning
RL	Reinforcement Learning
ROS	Robot Operating System
